# Different facets of religiosity and their longitudinal associations with psychotic-like experiences in the general population

**DOI:** 10.1007/s00127-025-02961-w

**Published:** 2025-07-08

**Authors:** Błażej Misiak, Julian Maciaszek

**Affiliations:** Department of Psychiatry, Wroclaw Medical University, Wroclaw, Poland

**Keywords:** Hallucinations, Delusions, Psychosis, Religious faith, Spirituality

## Abstract

**Purpose:**

Religiosity manifests in a variety of behaviors and activities that can be divided into intrinsic (IR), extrinsic organizational (EORG), and extrinsic non-organizational religiosity (ENORG). It has been shown that religiosity might be associated with the occurrence of psychotic-like experiences (PLEs). However, the understanding of this association might be limited due to a lack of longitudinal studies addressing the effects of various religiosity types on the occurrence of PLEs. The present study aimed to explore the longitudinal associations of religiosity dimensions with PLEs.

**Methods:**

A total of 5,099 general population individuals (aged 44.9 ± 15.4 years, 52.2% women) were assessed at baseline and reinvited for the follow-up assessment after 6– 7 months. Religiosity and PLEs were assessed using the Duke University Religion Index and Prodromal Questionnaire– Brief, respectively.

**Results:**

Individuals who completed assessments at both timepoints (n = 3,275) and non-completers (n = 1,824) did not differ significantly with respect to baseline characteristics. After adjustment for covariates (age, gender, the level of education, employment status, place of residence, social network size, substance use, psychiatric treatment history, depressive and anxiety symptoms), IR was bidirectionally associated with PLEs and related distress. Moreover, PLEs, together with associated distress, predicted higher levels of ENORG, but not its changes over time. However, observed associations showed small effect size estimates, especially in the case of ENORG. No significant associations were found for EORG.

**Conclusion:**

Findings from the study indicate complex and rather bidirectional associations of more intimate dimensions of religiosity with PLEs.

## Introduction

Psychotic-like experiences (PLEs) refer to subclinical phenomena marked by impairments of perception and unconventional beliefs, with the lifetime prevalence estimated at around 7% in the general population [1]. Recent insights stemming from a comprehensive meta-analysis indicate that roughly one-third of these individuals report persistent PLEs [2]. Despite their subclinical categorization, PLEs serve as a transdiagnostic indicator for the emergence of mental disorders that extend beyond the diagnostic boundaries of psychotic disorders [3]. Importantly, PLEs can be efficiently screened in various populations, often with the use of single assessment items [4], and there is evidence supporting the potential advantages of reducing or preventing their occurrence [5]. Thus far, a variety of factors contributing to the onset or persistence of PLEs have been documented. These factors include disadvantaged socioeconomic status, low educational attainment, cannabis use, and a history of childhood trauma [2, 6]. The clinical relevance of PLEs can already be observed in a short-term perspective. Indeed, studies with a relatively short observation period of 6 months have demonstrated that PLEs predict the intent to seek treatment regardless of co-occurring symptoms [7], suicide risk [8], and the emergence or progression of co-occurring psychopathology [9]. It has also been found that depressive symptoms and sleep disturbances might predict the occurrence of PLEs over a 6-month period [10, 11]. Taken together, these observations provide the rationale to investigate early trajectories of PLEs.

Cultural aspects might largely influence the way psychopathological symptoms are explained, experienced, and managed. Moreover, culture may impact causation of psychopathology [12]. There are several cultural determinants of mental health, among which an important role is played by religiosity. Individual religiosity and related activities are a complex and multidimensional construct that covers the level of religious beliefs as well as attitudes to religious principles and activities [13]. In general, these aspects are reflected by three dimensions of religiosity covering extrinsic organizational religiosity (EORG; i.e., church, temple, or institutional attendance), extrinsic non-organizational religiosity (ENORG; i.e., religion activities limited to more individual activities, such as praying, reading religious contents, and meditation), and intrinsic religiosity (IR; defined as subjective beliefs and motivation related to religiosity) [14]. Importantly, religiosity might show various trajectories over time and a greater speed of changes might be observed in some individuals [15, 16]. However, it remains largely unknown as to whether distinct trajectories of changes in religiosity might be associated with mental health characteristics [17].

To date, various aspects of religiosity have been investigated with respect to PLEs. The analyses of data from large epidemiological surveys revealed that higher levels of religiosity at various indicators are associated with higher odds of PLEs [18]. Another study demonstrated that the association of religiosity with PLEs depends on the religious affiliation [19]. Specifically, the authors found that the Christian affiliation is associated with lower odds of PLEs while the non-Christian or multiple-religion affiliations are associated with higher odds of PLEs. Interesting insights were also provided by the recent study in a predominantly Muslim society [20]. The authors found that ENORG is associated with experiencing reduced distress related to hallucinations, both directly and indirectly (i.e., through IR). In turn, EORG was associated with increased hallucinations distress or impact, only through higher levels of IR. Similarly, a greater EORG was associated with higher levels of hallucinations and perceptual abnormalities, while a greater IR was correlated with reduced suspiciousness in individuals at ultra-high risk of psychosis [21]. However, insights into the temporal ordering of religiosity and PLEs are limited as these studies were based on a cross-sectional design.

To date, only one study investigated longitudinal associations between religiosity and PLEs [22]. The authors demonstrated a non-linear relationship between religiosity and auditory vocal hallucinations among adolescents. Specifically, the study revealed that moderately religious adolescents are significantly more likely to develop auditory vocal hallucinations compared to their non-religious and strongly religious counterparts. Taking into consideration limited insights into longitudinal effects, the present study aimed to assess the directionality of associations between various categories of religiosity and PLEs in the general population sample in a short-term perspective. In general, previous studies have not clearly supported a specific direction of causality and suggested that specific facets of religiosity might be differentially associated with PLEs. Therefore, our hypotheses were twofold. First, we hypothesized bidirectional associations between religiosity and PLEs. Second, we assumed that EORG, ENORG, and IR show differential associations with PLEs.

**Methods**

*The cohort: *To develop the present cohort, the quota sampling method was used to provide sample representativeness with respect to sociodemographic characteristics (age, gender, education, employment status, and place of residence). All assessments were performed using an internet-based survey. The baseline assessment was scheduled between July and August, 2024. Participants who completed the baseline assessment were invited for the follow-up assessment in February, 2025. The cohort was developed by a research company using its own online access panel of registered and verified participants. The panel undergoes continuous development through regular campaigns. Underrepresented individuals (e.g., old-age individuals, ethnic and social minorities) are continuously enrolled through additional campaigns. For the present study, all panel members, aged at least 18 years, were considered eligible. They were informed about confidentiality of all assessments and provided online informed consent to participate. The study was approved by the Bioethics Committee at Wroclaw Medical University, Wroclaw, Poland (approval number: 553/2024).

**Measurements**

*Religiosity:* To measure the level of religiosity, the Duke University Religion Index (DUREL) was administered [23]. The DUREL is a brief measure that was developed to assess EORG (1 item), ENORG (1 item), and IR (3 items). The items referring to EORG and ENORG are rated on a 6-point Likert scale, while the IR items are based on a 5-point scale. The total DUREL score ranges between 5 and 27, where higher scores reflect a greater level of religiosity. It has good psychometric properties with respect to internal consistency, test-retest reliability, and convergent validity with other measures of religiosity [23, 25]. In the present study, the Cronbach's alpha of DUREL was 0.912 at baseline.*PLEs:* To assess PLEs, the Prodromal Questionnaire-Brief (PQ-B) was used [26, 27]. The PQ-B measures the level of 21 symptoms over a preceding month that are rated on two subscales, i.e., the presence subscale and distress subscale. The first one is based on yes-or-no responses while the second one is rated on a 5-point scale (1 -"strongly disagree", 5 -"strongly agree"). The total scores are 0 - 21 (the presence subscale) and 21 - 105 (the distress subscale). In this study, the Cronbach’s alphas for the PQ-B were 0.873 (the presence subscale) and 0.894 (the distress subscale) at baseline.

*Depressive symptoms:* To assess depressive symptoms, the Patient Health Questionnaire-9 (PHQ-9) was implemented [28]. It is based on 9 items measuring the frequency of specific depressive symptoms over preceding two weeks (potential responses range from 0 -"not at all"to 3 -"nearly every day"). The total PHQ-9 score ranges between 0 and 27 with higher scores corresponding with a greater severity of depressive symptoms. In the present cohort, the Cronbach's alpha of the PHQ-9 was 0.897 at baseline.

*Anxiety symptoms:* Anxiety symptoms were recorded using the Generalized Anxiety Disorder-7 (GAD-7) [29]. It includes 7 items (rated on 4-point scale: 0 -"not at all", 3 -"nearly every day") that refer to generalized anxiety symptoms in the preceding 2 weeks (a 4-point scale; 0 -"not at all", 3 -"nearly every day"). The total score is 0 - 21, where higher scores indicate a greater level of anxiety symptoms. In this study, the Cronbach's alpha of GAD-7 was 0.944 at baseline.

*Social network size:* To measure the social network size, the 6-item version of Lubben Social Network Scale (LSNS-6) was used [30]. The LSNS-6 items record the number of family members and friends who are seen or heard at least once a month, with whom the respondent can talk about private matters, and who can be called on for help (a 6-point scale). The total LSNS-6 score is 0 - 20. Higher scores correspond with a greater social network size. In this study, the Cronbach's alpha of the LSNS-6 was 0.876 at baseline

*Accuracy of responses: *To ensure reliability of responses, a number of accuracy measures were implemented during and after the survey. Respondents who failed any accuracy checks were not included in the final dataset. Specifically, they were excluded in the case of showing the following violations across accuracy checks: (1) short survey completion time (i.e., below 30% of the median completion time); (2) failure to pass attention checks (i.e., participants were asked to respond to items requesting them to select a specific answer); (3) inconsistent responses to repeated items, and (4) responses with random strings of characters.

### Data analysis

Only complete records were analyzed. The general characteristics, psychopathological symptoms, and religiosity of individuals who completed assessments at both timepoints and those who were not assessed at the follow-up were compared using the t-tests (continuous variables) and chi-square tests (categorical variables). Bivariate correlations across baseline measures of PLEs and religiosity were assessed using the Pearson’s correlation coefficients. Next, linear regression analyses were performed. To control for multicollinearity, the variance inflation factor (VIF) was assessed. The VIF scores lower than 4 were interpreted as showing the lack of multicollinearity [31]. First, the follow-up PQ-B presence and distress scores as well as their changes over time were included as dependent variables in separate models. The baseline scores of various religiosity categories (EORG, ENORG, and IR) represented independent variables. Next, the follow-up scores of EORG, ENORG, and IR as well as their changes over time were included as dependent variables in separate linear regression models. Independent variables were the baseline PQ-B presence and distress scores. The PQ-B presence and distress scores were analyzed separately as they showed multicollinearity while included together. All linear regression analyses accounted for the effects of age, gender, the level of education, employment status, place of residence, social network size, lifetime psychiatric treatment, substance use in the preceding month, depressive and anxiety symptoms. Results were interpreted as significant if the *p*-value was lower than 0.05.

## Results

The cohort included 5,099 individuals who completed the baseline assessment. The follow-up assessment was completed by 3,275 participants (64.2% of the initial sample). The following reasons for non-participation in the follow-up assessment were recorded: (1) non-response to invitations (*n* = 1,448); (2) the survey was initiated but was not completed (*n* = 187); (3) the failure of accuracy checks while completing the survey (*n* = 93), and (4) the failure of accuracy checks at the level of completed dataset (*n* = 96). Individuals who did not complete the follow-up assessment did not differ significantly from those who completed assessments at both timepoints with respect to the baseline sociodemographic characteristics, social network size, the level of religiosity, and symptom scores (Table 1).

**Table 1:** The general characteristics of cohort participants

	Total sample (*n* = 5,099)	Completers (*n* = 3,275)	Non-completers (*n* = 1,824)	*p*
Age, years	44.9 ± 15.4	45.2 ± 15.7	44.8 ± 15.2	0.189
Gender, men	2431 (47.7)	1568 (47.9)	863 (47.3)	0.718
Education Primary Vocational Secondary Higher	94 (1.8) 411 (8.1) 2098 (41.1) 2496 (49.0)	53 (1.6) 273 (8.3) 1345 (41.1) 1604 (49.0)	41 (2.2) 138 (7.6) 753 (41.3) 892 (48.9)	0.858
Employment Unemployed Retired/disability pension Student Part-time work Full-time work	435 (8.5) 1002 (19.6) 234 (4.6) 482 (9.5) 2946 (57.8)	265 (8.1) 632 (19.3) 156 (4.8) 317 (9.7) 1905 (58.1)	170 (9.3) 370 (20.3) 78 (4.3) 165 (9.0) 1041 (57.1)	0.406
Place of residence Rural Urban, < 50,000 inhabitants Urban, 50,000–150,000 inhabitants Urban, 150,000–500,000 inhabitants Urban, >500,000 inhabitants	1650 (32.4) 1166 (22.9) 816 (16.0) 714 (14.0) 753 (14.7)	1065 (32.5) 750 (22.9) 525 (16.0) 457 (14.0) 478 (14.6)	585 (32.1) 416 (22.8) 291 (16.0) 257 (14.0) 275 (15.1)	0.991
Monthly income < 750 USD 750–1500 USD 1,500–2,500 USD 2,500–3,750 USD > 3,750 USD Refused to answer	1082 (21.1) 2485 (48.7) 722 (14.2) 133 (2.6) 44 (0.9) 633 (12.4)	695 (21.2) 1621 (49.6) 468 (14.3) 80 (2.4) 31 (0.9) 380 (11.6)	387 (21.2) 864 (47.4) 254 (13.9) 53 (2.9) 13 (0.7) 253 (13.9)	0.170
Psychiatric treatment Lifetime Preceding month	1108 (21.7) 539 (10.6)	705 (21.5) 327 (10.0)	403 (22.1) 512 (28.1)	0.638 0.068
Substance use, preceding month^*^	353 (6.9)	213 (6.5)	140 (7.7)	0.353
PLEs (presence), PQ-B Baseline Follow-up	3.9 ± 4.2 –	3.8 ± 4.1 3.6 ± 3.9	4.0 ± 4.3 –	0.099 –
PLEs (distress), PQ-B Baseline Follow-up	8.0 ± 10.7 –	7.8 ± 10.6 7.5 ± 10.3	8.1 ± 11.0 –	0.071 –
Depressive symptoms, PHQ-9 Baseline Follow-up	7.4 ± 6.0 –	7.3 ± 5.8 7.0 ± 5.7	7.6 ± 6.1 –	0.078
Anxiety symptoms, GAD-7 Baseline Follow-up	6.1 ± 5.5 –	5.9 ± 5.3 5.7 ± 5.2	6.2 ± 5.6 –	0.068
Social network size, LSNS-6 Baseline Follow-up	15.9 ± 6.0 –	16.0 ± 6.5 15.7 ± 5.9	15.8 ± 5.8 –	0.228
EORG, DUREL Baseline Follow-up	2.8 ± 1.5 –	2.9 ± 1.5 2.9 ± 1.6	2.8 ± 1.5 –	0.089
ENORG, DUREL Baseline Follow-up	2.2 ± 1.7 –	2.3 ± 1.7 2.3 ± 1.7	2.2 ± 1.6 –	0.111
IR, DUREL Baseline Follow-up	8.4 ± 3.9 –	8.5 ± 4.0 8.5 ± 3.8	8.4 ± 3.8 –	0.142

Data are reported as mean ± SD or n (%)

If not specified, the data are reported for baseline assessment

^*^Except for nicotine and alcohol

*Note:* DUREL, the Duke University Religion Index; ENORG, extrinsic non-organizational religiosity; EORG, extrinsic organizational religiosity; GAD-7, the Generalized Anxiety Disorder-7; IR, intrinsic religiosity; LSNS-6, the Lubben Social Network Scale-6; PHQ-9, the Patients Health Questionnaire-9; PQ-B, the Prodromal Questionnaire– Brief

The bivariate correlations between baseline measures assessed in the present study are reported in Table 2. As expected, significant and positive correlations were found within the measures of psychopathological symptoms. Similarly, all categories of religiosity were significantly and positively correlated with each other. However, the measures of psychopathological symptoms showed significant, albeit weak, correlations with specific categories of religiosity.

**Table 2:** The bivariate correlations across baseline measures assessed in the present study

	1.	2.	3.	4.	5.	6.	7.
1. PLEs (P)	-						
2. PLEs (D)	0.926^c^	-					
3. DEP	0.521^c^	0.559^c^	-				
4. ANX	0.522^c^	0.564^c^	0.829^c^	-			
5. SNS	–0.156^c^	–0.174^c^	–0.266^c^	–0.232^c^	-		
6. EORG	–0.015	–0.008	–0.047^b^	–0.005	0.213^c^	-	
7. ENORG	0.042^a^	0.033	–0.002	0.036^a^	0.120^c^	0.651^c^	-
8. IR	0.052^b^	0.050^b^	–0.041^a^	0.008	0.189^c^	0.713^c^	0.648^c^

Pearson’s correlation coefficients are reported

^a^p < 0.050, ^b^p < 0.010, ^c^p < 0.001

*Note:* ANX, generalized anxiety symptoms; DEP, depressive symptoms; ENORG, extrinsic non-organizational religiosity; IR, intrinsic religiosity; EORG, extrinsic organizational religiosity; PLEs (D), psychotic-like experiences (related distress); PLEs (P), psychotic-like experiences (presence); SNS, social network size

The results of linear regression analyses testing for the effects of baseline religiosity on follow-up PLEs and their changes over time are shown in Table 3. A higher level of baseline IR was correlated with higher follow-up scores of PLEs and their greater progression over time before and after adjustment for covariates. These results were significant with respect to the presence of PLEs and related distress. Other categories of religiosity were not correlated with the scores of PLEs over time.

**Table 3:** The results of linear regression analyses testing for the correlations of baseline religiosity measures with follow-up measures of psychotic-like experiences and their changes over time

Independent variable	Dependent variable	Unadjusted model	*p*	Adjusted model	*p*
		β		β	
EORG	PLEs (presence)	–0.104	< 0.001	–0.024	0.297
ENORG		0.046	0.058	–0.002	0.923
IR		0.102	**< 0.001**	0.102	**< 0.001**
EORG	ΔPLEs (presence)	0.034	0.204	0.021	0.440
ENORG		–0.013	0.590	–0.005	0.830
IR		0.119	**< 0.001**	0.114	**< 0.001**
EORG	PLEs (distress)	–0.065	0.014	0.024	0.284
ENORG		0.033	0.173	0.037	0.112
IR		0.090	**< 0.001**	0.084	**< 0.001**
EORG	ΔPLEs (distress)	0.051	0.058	0.033	0.211
ENORG		–0.003	0.888	0.008	0.756
IR		0.098	**< 0.001**	0.095	**< 0.001**

Δ estimated by subtracting the baseline scores from the follow-up scores

Significant associations (*p* < 0.05) are bolded

^*^Adjusted for age, gender, education, employment status, place of residence, social network size, substance use, lifetime history of psychiatric treatment, depressive and anxiety symptoms

*Note:* ENORG, extrinsic, non-organizational religiosity; EORG, extrinsic, organizational religiosity; IR, intrinsic religiosity; PLEs, psychotic-like experiences

The effects of baseline PLEs on follow-up measures of religiosity and their changes over time are reported in Table 4. Higher baseline scores of PLEs on both subscales (i.e., the presence and distress subscales) predicted higher follow-up levels of IR and its changes over time in adjusted and unadjusted analyses. Similarly, higher baseline scores of PLEs on both subscales predicted higher levels of follow-up ENORG, but not its changes over time. However, these effects were significant only after adjustment for covariates.

**Table 4:** The results of linear regression models testing for the correlations of baseline psychotic-like experiences with follow-up measures of various religiosity facets and their changes over time

Independent variable	Dependent variable	Unadjusted model		Adjusted model	
		β	*p*	β	*p*
PLEs (presence)	EORG	–0.014	0.409	0.012	0.542
	ΔEORG	–0.001	0.998	–0.006	0.776
	ENORG	0.030	0.091	0.045	**0.030**
	ΔENORG	–0.019	0.274	–0.013	0.543
	IR	0.051	**0.003**	0.083	**< 0.001**
	ΔIR	0.089	**0.001**	0.084	**0.012**
PLEs (distress)	EORG	–0.003	0.868	0.034	0.107
	ΔEORG	0.011	0.516	0.010	0.638
	ENORG	0.028	0.109	0.046	**0.033**
	ΔENORG	–0.008	0.644	0.005	0.831
	IR	0.049	**0.005**	0.086	**< 0.001**
	ΔIR	0.046	**0.013**	0.053	**0.009**

Δ estimated by subtracting the baseline scores from the follow-up scores

Significant associations (*p* < 0.05) are bolded

^*^Adjusted for age, gender, education, employment status, place of residence, social network size, substance use, lifetime history of psychiatric treatment, depressive and anxiety symptoms

*Note:* ENORG, extrinsic, non-organizational religiosity; EORG, extrinsic, organizational religiosity; IR, intrinsic religiosity; PLEs, psychotic-like experiences

## Discussion

Main findings from the present study indicate that the correlation of religiosity with PLEs shows some complexity across its distinct dimensions. Specifically, IR might show bidirectional correlations with PLEs. In turn, ENORG, although weekly correlated, should rather be interpreted as a potential consequence of PLEs. The correlations of IR and ENORG with PLEs were also observed at baseline; however, only IR predicted progression of PLEs and associated distress over time. Notably, baseline PLEs were not found to predict changes of religiosity over time. Altogether, the findings posit that more intimate aspects of religiosity, covered by ENORG and IR, but not EORG, are related to the occurrence of PLEs.

One perspective of our findings is that a greater IR might predict the occurrence of PLEs. The largest, albeit cross-sectional, analysis addressing the association between religiosity and PLEs across 18 countries revealed that having a religious affiliation is not associated with PLEs [18]. However, the authors found that five indices of religious activity (i.e., considering religion as very important when growing up, or in daily life, often seeking comfort in religion when experiencing problems, often thinking about religion to help with decision making in daily life) are associated with increased odds of PLEs among individuals reporting religious affiliation [18]. Although these indices refer to various dimensions of religiosity, some of them might refer to IR, e.g., seeking comfort in religion when experiencing problems and thinking about religion to help with decision making. Interestingly, the measures of EORG (i.e., the frequency of attendance at religious services) were not associated with the odds of PLEs which is consistent with our findings. Another cross-sectional analysis of data collected across 140 college campuses in the United States revealed that religious importance is associated with increased odds of PLEs only among individuals self-identified as Atheists, Agnostics, and Buddhists, those reporting a lack of a particular affiliation, and participants with multiple religious affiliations [19]. Interesting insights suggesting causal associations between religiosity and religious delusions were provided by Anderson-Schmidt et al. [32]. The authors found that strong religious activity and a high polygenic schizophrenia risk score are independent risk factors for the occurrence of religious delusions among individuals with schizophrenia or schizoaffective disorder.

The second perspective of our observations is that PLEs might predict increases of religiosity over time, especially with respect to IR. This perspective suggests that some individuals with PLEs might turn to religion in order to cope with distressing experiences. In general, turning to religion is perceived as an adaptive coping strategy that acts through reappraisal and strengthening self-efficacy [33]. Turning to religion in the context of psychotic symptoms is often perceived as the way of coping that allows to maintain quality of life and a better outcome [34, 35]. Moreover, there is evidence that the extent of finding comfort in religious beliefs and practices is positively correlated with the quality of social functioning [36]. Recent findings from a non-clinical sample of individuals representing a predominantly Muslim society also demonstrated that EORG and ENORG might be correlated with lower levels of distress related to experiencing hallucinations and better daily functioning [20]. Simultaneously, this study revealed that higher levels of IR are correlated with worse daily functioning and a greater distress related to hallucinations. Of note, there are several methodological differences between our study and the one by Khaled et al. [20]. First, our study was performed in the Polish population, where the vast majority of citizens declare the Roman Catholic religious affiliation. Second, the study by Khaled et al. [20] used a cross-sectional design, thereby limiting insights into temporal ordering of PLEs and religiosity.

However, less adaptive mechanisms behind turning to religion should also be considered. It might be hypothesized that individuals with paranoia-like beliefs may be more willing to engage in religious practices, because belonging to the community dedicated to a single idea or belief can, on one hand, reduce their sense of persecution threat [37]. On the other hand, from their perspective, religious coping may stay in harmony with a dualistic, paranoic view of the world dominated by two forces, i.e., the good one (“us”) and the one representing a threat (“them”), in accordance with the psychodynamic projection defense mechanism [38]. In agreement with these considerations, paranoia has been associated with an increased perception of hostility and culpability in scenarios characterized by social ambiguity [39].

Findings from the present study should be interpreted in the context of certain limitations. It is important to note that observed effect size estimates, although significant, were small and thus the relevance of associations between religiosity and PLEs should be interpreted with caution. This might be of particular relevance for ENORG. However, these findings are in agreement with previous population-based studies that also reported relatively small effect size estimates [18, 19]. Another limitation is related to the fact that our cohort was non-clinical and did not include a comprehensive clinical assessment. Therefore, the potential to translate findings into clinical contexts might be limited. It should further be pointed out that a relatively short observation period and two waves of assessment may not be sufficient to detect notable changes across processes related to individual religiosity. Furthermore, it is important to note that our study did not provide insights into the effects of specific religious affiliations. However, despite an ongoing secularization, the majority of Polish inhabitants still declare the Christian affiliation [40]. Also, we believe that the findings might be informative as the questionnaire used to assess religiosity does not cover affiliation-specific contexts. Another limitation of the study is the overrepresentation of highly educated participants, which may affect the generalizability of findings. Additionally, our sample predominantly included middle-aged individuals, while previous studies have shown higher prevalence rates of PLEs in adolescents and young adults [41, 43]. Finally, the study, although longitudinal, should be interpreted as an observational one and thus it informs about temporal ordering of variables rather than causal effects.

In sum, findings from the present study indicate that religiosity and PLEs show bidirectional associations. In this regard, religiosity needs to be interpreted both as a range of processes that might shape the vulnerability to mental disorders and the way individuals cope with distressing symptoms. It is also important to recognize the complexity of associations between religiosity and PLEs, where more intimate processes (IR and ENORG), rather than explicitly organizational religious activities (EORG), are related to their occurrence. Although the findings are limited to a non-clinical context, they tentatively suggest that an individualized approach to assessing religiosity might be worth considering in case conceptualization. Indeed, it might be necessary to assess the role of specific aspects of religiosity as its various dimensions may play differential roles in individual cases. From the research perspective, the findings provide grounds for larger, collaborative, and international studies investigating observed associations across various religious affiliations, cultural norms, and clinical contexts. Future studies also need to investigate the longitudinal associations of religiosity with vulnerability to mental disorders in various clinical and non-clinical populations. From the general population perspective, it is important to investigate as to whether various sociodemographic characteristics moderate these associations.

## Data Availability

Data supporting the present study are available from the corresponding author upon a reasonable request.
